# Assessment of methicillin resistant *Staphylococcus* Aureus detection methods: analytical comparative study

**DOI:** 10.11604/pamj.2017.27.281.9016

**Published:** 2017-08-21

**Authors:** Omer Mohammed Ali Ibrahim, Naser Eldin Bilal, Omran Fadl Osman, Magzoub Abbas Magzoub

**Affiliations:** 1 Department of Microbiology, Faculty of Medicine and Health Sciences, University of El-imam El-mahdi P.O Box 209, Kosti City, Sudan; 2 Department of Medical Microbiology, Faculty of Medical Laboratory Sciences, Khartoum University P.O. Box 11081, Khartoum, Sudan; 3 Faculty of Sciences, Khartoum University P.O. Box 11081, Khartoum, Sudan; 4 Department of Medical Laboratories, College of Applied Sciences, Qassim University P.O Box 1083 Burayda, Saudi Arabia

**Keywords:** MRSA, oxacillin disc test, Staphylococcus aureus, latex agglutination sensitivity

## Abstract

**Introduction:**

The heterogeneous expression of methicillin resistance in *Staphylococcus aureus* (MRSA) affects the efficiency of tests available to detect it. The objective of this study was to assess four phenotypic tests used to detect MRSA.

**Methods:**

This is an analytical comparative study conducted among sudanese patients during period from May 2012 to July 2014, *Staphylococcus aureus* strains were isolated and identified by conventional methods, and then confirmed by PCR detection of coagulase gene. PCR detection of mecA gene was used as a gold standard to assess oxacillin resistance screen agar base (ORSAB), oxacillin disc, cefoxitin disc (at different temperatures and incubation periods) and MRSA-latex agglutination test. *S.aureus* ATCC 25923 was used as control. Sensitivity and specificity were calculated.

**Results:**

MRSA- latex agglutination was the most accurate test; it showed 100% of both sensitivity and specificity, followed by cefoxitin disc with sensitivity of 98.48% and specificity of 100%. However, both of oxacillin disc and oxacillin resistance screen agar base showed less accurate results, and were affected by incubation periods. Oxacillin disc after 24 h incubation both at 30°C and 35°C showed sensitivity and specificity values of 87.88% and 96.23%, respectively. However, after 48h incubation the test at 30°C showed sensitivity and specificity values of 89.39%, and 94.34%, respectively. At 35°C (48h) it showed values of 89.39%, 92.45% respectively. Specificity of ORSAB was more than oxacillin disc at 35°C after 24h incubation 98.11% and 96.23%, respectively.

**Conclusion:**

MRSA- latex agglutination and cefoxitin disc diffusion tests are recommended for routine detection of MRSA.

## Introduction

Methicillin resistance in staphylococci is primarily due to the acquisition of a mobile staphylococcal chromosomal cassette which carries the *mec*A-gene, known as SCCmec [1]. This *mec*A-gene encodes an altered PBP, PBP2a (or PBP2’) [2]. The affinity of beta-lactams towards PBP2a is much lower than towards native PBP2, thus allowing continuous cell wall assembly [3]. Many clinical MRSA isolates express resistance to methicillin heterogeneously. This means that the majority of cells are susceptible to low concentrations of methicillin, and only a minority of cells can grow at high concentrations [4]. Reliable microbiological diagnosis of MRSA is essential for treatment, surveillance and control. Clinical microbiology laboratories play a central role in the detection, identification, antibiotic susceptibility testing, and confirmation of MRSA. Conventional laboratory detection of MRSA includes culturing the specimen, confirmation of *S. aureus* with identification tests, antimicrobial susceptibility testing, and finally, verification of MRSA, usually with molecular methods. This may take several days. Rapid diagnostic testing for MRSA directly from specimens allows the infected patient to obtain a more rapid verification of antimicrobial therapy, leading to a decrease in mortality, a reduction in vancomycin usage, shorter stays in hospital, and lower hospital costs [5]. Rapid tests are still more expensive than conventional ones, and not all laboratories are able to use them for financial reasons. Regardless of the diagnostics methods used, concomitant cultures are necessary to recover the organism for further antimicrobial susceptibility testing and for epidemiological typing [4]. The determination of methicillin resistance is importance in prognosis of *S. aureus* infection. The phenotypic detection methods are cheaper than genotypic but they are dependent up on environmental conditions. Therefore, the golden methods for detection of MRSA were Polymerase Chain Reaction (PCR) through detection of the *mec*A gene or its product latex agglutination. However, the limitation of PCR to the reference labs leads the latex agglutination methods to be the best predictor to detect MRSA [6]. Therefore the objective of this study was to investigate the performances of four phenotypic tests used to detect MRSA namely cefoxitin disc, oxacillin disc, oxacillin resistance screen agar base (ORSAB) test (at different temperatures and incubation periods), and MRSA- latex agglutination test, comparing them with the performances of gold standard PCR for detection of *mec* A gene.

## Methods

### *S. aureus* isolates

One hundred and nineteen of *Staphylococcus aureus* (*S.aureus*) isolates (66 mecA gene positive and 53 mecA gene negative) were included in this study .The isolates were from skin infections and anterior nares that were collected from patients at Kosti and Rabak hospitals, Sudan during the period from May 2012 through July 2014. Isolates were identified as *S.aureus* using conventional tests and confirmed by PCR detection of coagulase gene. Then they were tested for presence or absence of *mec*A gene.

### DNA extraction for amplification

G-spinTM Genomic DNA Extraction Kit was used for genomic DNA extraction. The kit designed for rapid isolation of genomic DNA, uses advanced silica-gel membrane technology column for rapid and efficient purification of genomic. Buffer system was optimized to allow rapid and simple cell lysis followed by selective binding of DNA to the column. The DNA extraction was performed according to the manufacturer instructions.

### Coagulase gene (coa) amplification

PCR amplification of the coagulase (coa) gene was done using (iNtRON´s Maxime PCR PreMix) master mix. The kits components in one tube for 1 reaction PCR, were Taq DNA polymerase 2.5U, dNTPs 2.5mM each, Buffer 1x and Gel Loading buffer 1x. The components were dissolved in distilled water into the tubes to a total volume of 20μl including volume of the DNA template 2μl, and primers (75 P moles) 0.75μl for each of the primer. The forward primer 5'ATA GAG ATG CTG GTA CAG G3' and the reverse primer 5'GCT TCC GAT TGT TCG ATG C3'. were used. Cycling takes place on a Thermal cycler following an initial denaturation at 94°C for 45 s. The cycling proceeded for 30 cycles of 94°C for 20 s, 57°C for 15 s, and 70°C for 15 s with a final step at 72°C for 2 min. The PCR products (5μl) were analyzed using horizontal 1.5% agarose gel electrophoresis using tris- borate EDTA buffer [7].

### MRSA detection by PCR to look for presence of mecA gene

PCR was done using (*iNtRON´s Maxime* PCR PreMix) master mix. The kits components in one PCR tube for 1 PCR reaction, were *Taq* DNA Polymerase 2.5U, dNTPs 2.5mM each, Buffer 1x and Gel Loading buffer 1x. The components were dissolved in distilled water into the tubes to a total volume of 20μl including volume of the DNA template 2μl and primers (20 P moles) 2μl for each of the primers. The forward primer corresponds to nucleotides 1282 to 1303(5´ AAAATCGATGGTAAAGGTTGGC) and the reverse primer complementary to nucleotides 1793 to 1814 (5´AGTTCTGC AGTACCGGATTTGC) were used. Cycling takes place on a Thermal cycler following an initial denaturation at 94°C for 45 s. The cycling proceeded for 30 cycles of 94°C for 20 s, 57°C for 15 s, and 70°C for 15 s with a final step at 72°C for 2 min. The PCR products (5μl of each) were analyzed using horizontal 1.5% agarose gel electrophoresis and tris- borate-EDTA buffer [7].

### Assessment of MRSA resistance detection methods

PCR detection of *mec*A gene was used as gold standard to assess ORSAB (at 35ºC for 24 h and 48 h), oxacillin disc test (at 30ºC and 35ºC for 24 h and 48 h) cefoxitin disc diffusion test (at 30ºC and 35ºC for 24 h and 48 h) and MRSA- latex agglutination test. *S. Aureus* ATCC 25923 was used as control.

### MRSA detection by Oxacillin Resistance Screen Agar Base (ORSAB) test

Ten microlitres a 0.5 McFarland standard suspension of *S.aureus* isolates were inoculated onto ORSAB (Oxoid). It is a nutritious and selective medium containing a high salt concentration and lithium chloride to suppress non-staphylococcal growth with mannitol and aniline blue for the detection of mannitol fermentation. It was supplemented with 2mg/l oxacillin to suppress MSSA and 50,000IU/l of polymyxin B to suppress Gram negative bacteria. Plates were incubated at 35°C, and examined after 24h and 48h, *S. aureus* was considerd MRSA when revealed growth on ORSAB as dark blue colonies due to fermentation of mannitol.

### MRSA detection by cefoxitin and oxacillin disc diffusion methods

Susceptibility of *S. aureus* isolates were tested against cefoxitin (30μg), oxacillin (1μg), by the (CLSI) agar disc diffusion method [8]. Suspensions of overnight *S. aureus* cultures were adjusted to turbidity of 0.5 McFarland standard. Swabs were dipped in suspensions and streaked evenly onto Mueller-Hinton agar (MHA) and left for few minutes to dry. Discs were applied aseptically on the plates. Two sets of plates were used for each of antibiotics one set was incubated at 30Cº and the other at 35ºC.The results were reported after two periods firstly after 24h and after 48h of incubation. Isolates were considered MRSA when the inhibition zone diameter was ≤ 10 mm for oxacillin, ≤ 21 mm for cefoxitin.

### RSA- latex agglutination test

MRSA-latex agglutination test (Oxoid PBP2´) was performed according to the manufacturer instructions. For each strain, a sterile 5μl loop was used to remove sufficient growth of *S. aureus* colonies grown on MHA to fill the internal diameter of the loop suspended in four drops of extraction reagent 1 into a microcentrifuge tube. The suspension was boiled for 3 minutes then the microcentrifuge tube was allowed to cool to room temperature and one drop of extraction reagent 2 added and mixed well. The mixture was centrifuged at 4500 rpm for 5 minutes. A 50 μl of the supernatant was added to each of the test circle and the control circle on a disposable test card and mixed with one drop of the test latex (anti-PBP 2a monoclonal antibody sensitized latex) and one drop of the negative control latex, respectively. The contents on the card was then mixed and rocked for 3 minutes and examined for presence or absence of agglutination. The result was recorded as positive, negative or weakly positive.

**Ethical consideration:** The study received ethical clearance from the Ethical Research Committee at the Faculty of Medicine, U of K.

## Results

### Assessment of MRSA resistance detection methods results

In this study, 119 isolates (66 *mec*A gene positive and 53 *mec*A gene negative), were investigated by four different MRSA detection methods namely cefoxitin disc, oxacillin disc, ORSAB, and MRSA- latex agglutination tests and compared with the gold standard PCR for detection of *mec* A gene. Cefoxitin and oxacillin discs tests were performed at different temperatures and incubation periods. Cefoxitin disc results were stable at different temperatures at 30°C and 35°C and incubation periods for 24h and 48h. Oxacillin disc showed variable results at different temperature at 30°C and 35°C and different incubation periods for 24h and 48h. ORSAB test was assessed at 35°C for 24h and 48h and showed variable results too. MRSA- latex agglutination test was the most accurate method that yielded 100% agreement with the gold standard PCR method. The sensitivity, specificity, negative and positive predictive values of those tests are illustrated in Table 1. MRSA- latex agglutination showed the highest sensitivity (100%) and specificity (100%) followed by cefoxitin disc with sensitivity of 98.48% and specificity of 100%. However, oxacillin disc and ORSAB tests were less stable and were affected largely by incubation periods; 48h incubation decreased their specificity and increased their sensitivity. Oxacillin disc at 30ºC after 48h slightly more specific than oxacillin disc at 35ºC for 48h incubation. Oxacillin disc for 24 h incubation both at 30°C and 35ºC showed sensitivity and specificity values of 87.88% and 96.23%, respectively. However, after 48h incubation the results of oxacillin disc varied, the test at 30°C showed sensitivity and specificity values of 89.39%, 94.34%. At 35°C (48h) it showed values of 89.39%, 92.45% respectively, hence it comes to say that the sensitivity has been increased while the specificity is decreased. Similar observation was noted with ORSAB test which showed sensitivity and specificity at 35°C for 24h 84.85% and 98.11%, respectively, and after 48h the values were 89.39% and 88.68%, respectively. The specificity of ORSAB was more than oxacillin disc at 35°C after 24h incubation 98.11% and 96.23%, respectively.

**Table 1:** Comparison between methicillin resistance detection methods and gold standard *mec*A PCR method in a total of 119 *S.aureus*strains( 66 *mec*A positive and 53 *mec* A negative)

Detection method	Tested *S. aureus* strains 119 isolates				Parameters			
*mec*A PCR method (Standard)	*mec*A positive (true resistant) 66	FS0	*mec* A negative (true susceptible) 53	FR0	Sensitivity (%)100	Specificity (%) 100	PPV%100	NPV%100
PBP2a Latex agglutination	66	0	53	0	100	100	100	100
Cefoxitin 30μg disc at 30°C^◦^ for 24 h	65	1	53	0	98.48	100	100	98.15
Cefoxitin 30μg disc at 30°C^◦^ for 48 h	65	1	53	0	98.48	100	100	98.15
Cefoxitin 30μg disc at 35°C for 24 h	65	1	53	0	98.48	100	100	98.15
Cefoxitin 30μg disc at 35°C for 48 h	65	1	53	0	98.48	100	100	98.15
Oxacillin1μg disc at 30°C for 24 h	58	8	51	2	87.88	96.23	96.67	86.44
Oxacillin1μg disc at 30°C for 48 h	59	7	50	3	89.39	94.34	95.16	94.34
Oxacillin1μg disc at 35°C for 24 h	58	8	51	2	87.88	96.23	96.67	86.44
Oxacillin1μg disc at 35°C for 48 h	59	7	49	4	89.39	92.45	93.65	87.5
^+^ORSAB at 35°C for 24 h	56	10	52	1	84.85	98.11	98.25	83.87
ORSAB at 35°C for 48 h	59	7	47	6	89.39	88.68	90.77	87.04

FS: False susceptible, FR: False resistant, PPV: Positive predictive value, NPV: Negative predictive value

+ Containing 2mg/l oxacillin and 50,000IU/l of polymyxin B.

## Discussion

A number of methods were recommended by CLSI, for the detection of MRSA. These methods except for PCR technique are prone to errors due to heterogeneous nature of methicillin resistance and dependence on environmental conditions. Detection of the *mec*A gene is the most reliable method for identification of MRSA isolates. However not all laboratories include molecular biology techniques in their routine clinical practice. For this reason, it is essential that phenotypic techniques able to detect MRSA isolates in a rapid and accurate manner are made available. In this study four different MRSA detection methods namely cefoxitin disc, oxacillin disc, ORSAB test and MRSA- latex agglutination test were assessed with the gold standard PCR for detection of *mec* A gene. MRSA- latex agglutination test was the most accurate method with 100% agreement with PCR method (Table 1). This result is in agreement with Louie et al. [9], Lee et al.[10] and Sangeetha et al. [11] who reported that both sensitivity and specificity of MRSA- latex agglutination were 100% compared with PCR for detection of *mec*A. However, Alipour and his colleagues [12] reported less sensitivity for MRSA- latex agglutination (97.29%) instead of (100%). This is in part may be because these studies methods have included different strains, which may differ significantly in heterogeneity, and behave differently under particular test conditions. Isolates producing small amounts of PBP2a may give weak agglutination reactions or agglutinate slowly. The Latex agglutination method is rapid and requires no special equipment and can be the best predictor to detect MRSA but it is expensive.

In this study, cefoxitin disc diffusion test showed maximum comparability of results with MRSA Latex agglutination than oxacillin disc diffusion and ORSAB (Table 1). It showed stable results at different temperatures (30°C and 35ºC) and incubation periods (24h and 48 h), with high sensitivity (98.48%), and specificity (100%). Cefoxitin disc diffusion method is a cheap and simple test. Accordingly, the cefoxitin disc diffusion test was found to be the method of choice to identify MRSA. This result is in agreement with previous studies about the suitability of cefoxitin disc diffusion test for MRSA identification [11, 13–15]. It was reported that disc diffusion with cefoxitin is more reliable than that with oxacillin [16]. It is a more potent inducer of the *mec*A gene with no special requirements of temperature or medium [17].

In this study, oxacillin disc diffusion test showed variable results at different temperature (30ºC and 35ºC) and at different incubation periods (24h and 48h). Sensitivity and specificity of oxacillin disc for 24 h incubation both at 30ºC and 35ºC were 87.88% and 96.23%, respectively. However, after 48h incubation the results of oxacillin disc varied, at 30ºC (48h) the test sensitivity and specificity values were 89.39% and 94.34%, ,respectively. At 35ºC (48h) the test showed values of 89.39% and 92.45%, respectively. Therefore more incubation increases the sensitivity and decreases the specificity of the test. A similar observation has been seen with ORSAB test; at 35ºC for 24h values of sensitivity and specificity were 84.85% and 98.11%, respectively. After 48h incubation the values were 89.39% and 88.68%, respectively. This may be due to the fact that PBPa production is induced by growth in the presence of beta lactam antibiotics such as oxacillin and cefoxitin. However, cefoxitin is assumed to be a better inducer of *mec*A gene expression than is oxacillin and thus, is better for screening heterogeneous MRSA populations expressing the *mec*A variable. That is to say oxacillin needs more time to express resistance. One study that included heterogeneous strains found that oxacillin disc diffusion method had low sensitivity [18]. The variation in results of oxacillin disc diffusion test and ORSAB test (both used oxacillin antibiotic) may be due to the difference in culture media where MHA was used for oxacillin disc test and ORSAB agar was used for ORSAB test.

## Conclusion

MRSA- latex agglutination and cefoxitin disc diffusion tests are preferable for detection of MRSA as routine methods.

### What is known about this topic

- The heterogeneous expression of methicillin resistance in *Staphylococcus aureus* (MRSA) affects the efficiency of phenotypic tests available to detect it, heteroresistance strains may vary with different areas in the world;
- Detection of the *mec*A gene is considered as the reference method for determining resistance to methicillin;
- From different parts of the world several studies showed contradictory recommendations on the accuracy of oxacillin resistance screen agar base (ORSAB), oxacillin disc, cefoxitin disc and MRSA-latex agglutination test.

### What this study adds

- To our knowledge it is the first time to asses those tests in Sudan so the assessment process included new strains from new area;
- The study showed that cefoxitin disc diffusion test is not affected by different temperature (30°C and 35°C) or incubation periods (24h and 48h), and it is concluded that cefoxitin disc is essential and, more useful screening method, that it should be incorporated into routine clinical practice because many laboratories throughout the world including Sudan do not have the capacity to develop molecular techniques or even MRSA- latex agglutination test for detecting MRSA isolates.

## Competing interests

The authors declare no competing interest.
